# Effects of Yeast Polysaccharide on Growth and Flavonoid Accumulation in *Fagopyrum tataricum* Sprout Cultures

**DOI:** 10.3390/molecules171011335

**Published:** 2012-09-25

**Authors:** Gang Zhao, Jianglin Zhao, Lianxin Peng, Liang Zou, Jingbo Wang, Lingyun Zhong, Dabing Xiang

**Affiliations:** College of Biological Industry, Chengdu University, Chengdu 610106, Sichuan, China

**Keywords:** buckwheat sprouts, flavonoids, *Fagopyrum tataricum*, yeast polysaccharide (YPS), elicitation

## Abstract

The purpose of this study was to investigate the effects of yeast polysaccharide (YPS) on growth and flavonoid accumulation in sprout cultures of *Fagopyrum tataricum* (tartary buckwheat). Without obvious change in the appearance of the sprouts, the exogenous YPS notably stimulated the production of functional metabolites in *F. tataricum* sprouts, and the stimulation effect was concentration-dependent. With 400 mg/L of YPS applied to the sprout cultures on day 6, the total rutin and quercentin content was effectively increased to 42.8 mg/gdw, or about 1.4-fold in comparison with the control of 31.2 mg/gdw. Feeding with 800 mg/L of YPS on day 9, the sprouts biomass was increased by about 8% compared to the control culture (0.99 gdw/100 sprouts *versus* 0.92 gdw/100 sprouts). Moreover, the present study revealed that the accumulation of these bioactive metabolites resulted from the stimulation of the phenylpropanoid pathway by YPS treatment. It could be an effective strategy for improving the functional quality of the *F. tataricum* sprouts provided with YPS.

## 1. Introduction

**Figure 1:**
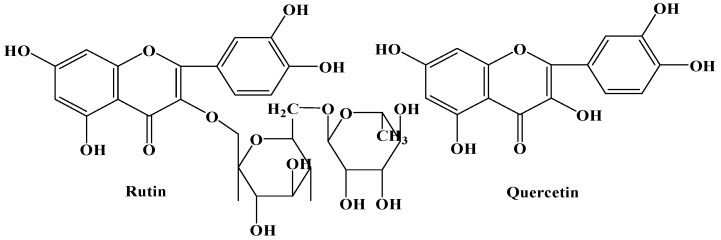
Chemical structures of rutin and quercetin.

Tartary buckwheat (*Fagopyrum tataricum* (L.) Gaertn) (Polygonaceae), a popular edible and medicinal crop mainly distributed in the southwest of China, northern India, Bhutan and Nepal, has been widely used as a daily diet and traditional medicine for a long time [1,2,3]. It is rich in vitamins, minerals, proteins, amino acids, dietary fiber, trace elements and various bioactive phytochemicals, and this has attracted many researchers’ attention. The major functional components of *F. tataricum* have been demonstrated to be flavonoids such as rutin and quercetin (Figure 1). Recent studies have revealed that these bioactive compounds had notable antioxidant, hypocholesterolemic, antidiabetic, antimicrobial, and antitumor activities, and were beneficial for human health [4,5,6,7]. Currently, there are a large number of buckwheat-based food products available on the market such as buckwheat flour, noodles, bread, tea, vinegar and sprouts [8,9,10].

Buckwheat sprouts are traditional used as a nutritional and health food. They have a soft and slightly crispy texture and an attractive fragrance [11,12]. In recent years, buckwheat sprouts have been under the spotlight in the international market due to their high nutritional value and health functions. Through sprouting, the buckwheat sprouts are more nutritious than their seeds. In particular, the contents of the major functional phytochemicals (*i.e.*, rutin, quercetin and γ-aminobutyric acid), which have a variety of interesting pharmacological effects, are also effectively increased [11,12,13,14]. 

Nowadays, bioactive phytochemicals have become one of the major research topics in the field of functional foods. Lots of research has focused on developing efficient strategies for enhancing the production of useful metabolites in edible plants without gene modification or breeding [15]. As the biosynthesis of many secondary metabolites in plants is usually a defense response of plants to biotic and abiotic stresses, their accumulation can be effectively stimulated by biotic and abiotic elicitors, making elicitation is one of the most effective strategies for improving bioactive secondary metabolite production in plant tissue cultures [16,17,18]. The most common and effective elicitors used in previous studies mainly include the components of microbial cells, especially poly- and oligosaccharides (biotic), and heavy metal ions, UV radiation, and hyperosmotic stress (abiotic), and the signaling molecules in plant defense responses such as salicylic acid (SA) and methyl jasmonate (MJ). Kim *et al*. first reported that the accumulation of functional metabolites (rutin, orientin, isoorientin and vitexin) in *Fagopyrum esculentum* sprouts was effectively stimulated by exogenous MJ, and their nutritional quality was also successfully improved [15].

Yeast polysaccharide (YPS) has been regarded as an efficient biotic elicitor for stimulating secondary metabolite production in plant cell and tissue culture. Production of many valuable bioactive compounds (*i.e.*, artemisinin, azadirachtin, plumbagin, tanshinones) has been successfully stimulated by YPS elicitors [19,20,21,22,23]. To the best of our knowledge, there were no previous reports about the effects of YPS on functional metabolite accumulation in the sprout cultures of *F. tataricum*. Therefore, we carried out a research program to investigate the effects of YPS on the growth and flavonoids production in *F. tataricum* sprouts. Additionally, the intracellular phenylalanine ammonia lyase (PAL) activity of *F. tataricum* sprout cells treated by YPS was examined, and the potential relationship to plant stress response was also discussed. 

## 2. Results and Discussion

### 2.1. Sprout Growth and Flavonoids Accumulation of F. tataricum

**Figure 2:**
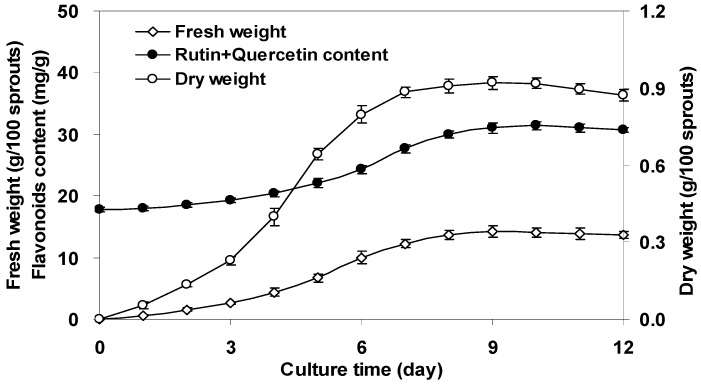
Time courses of biomass and flavonoids accumulation in the sprout cultures of *F. tataricum*. The error bars represented standard deviations, (*n* = 3).

The time course of *F. tataricum* sprout growth exhibited a slow growth or lag phase period in the first 2 days, a rapid, linear growth period between days 3–7, and a stationary or declining phase in the subsequent days, reaching the maximum biomass concentration (14.20 gfw/100 sprouts) around day 9 (Figure 2). Correspondingly, the maximum biomass concentration of 0.92 gdw/100 sprouts was obtained on day 9. As for flavonoid accumulation, the content of major flavonoids (rutin and quercetin) in *F. tataricum* sprouts remained at a very low level from days 1–5, and then increased steadily from days 6–10 to reach a highest content of 31.4 mg/gdw. These results indicated that day 10 was a suitable time for harvesting *F. tataricum* sprouts with respect to their high biomass concentration and flavonoids yield.

### 2.2. Effects of YPS on F. tataricum Sprout Growth and Flavonoids Production

**Figure 3:**
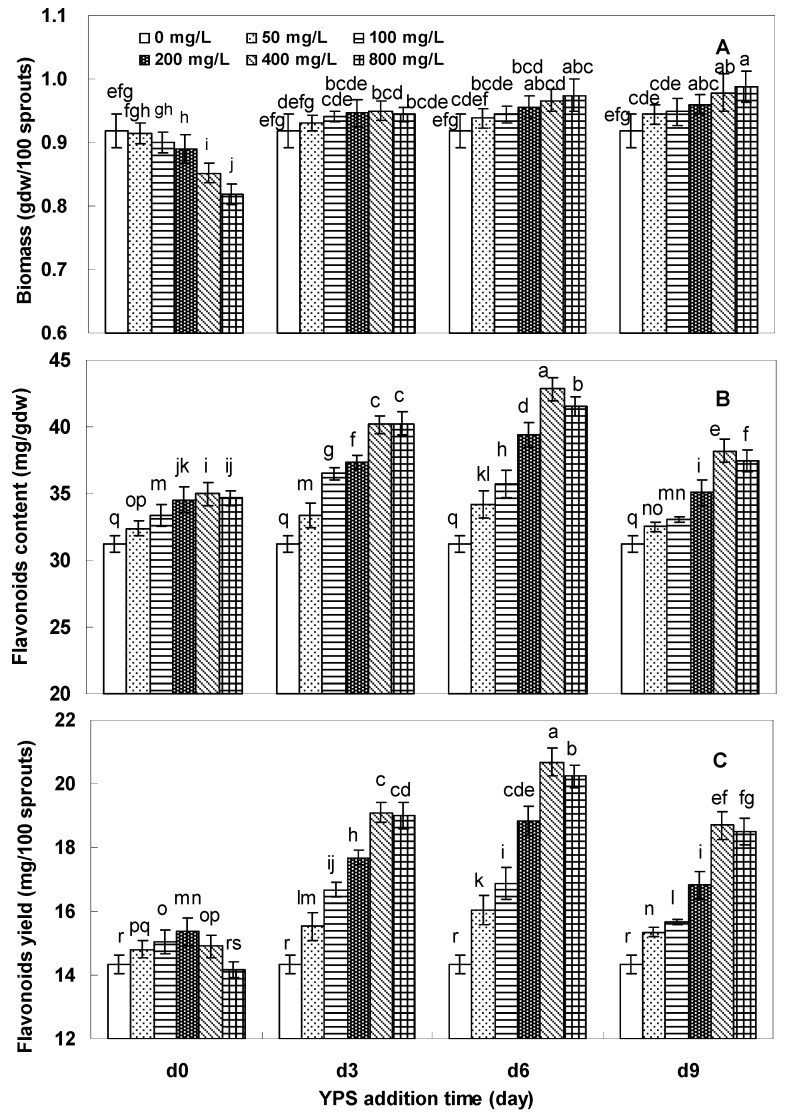
Effects of YPS (0, 50, 100, 200, 400 and 800 mg/L) on the sprout biomass (**A**), flavonoids content (**B**), and flavonoids yield (**C**) of *F. tataricum* sprout cultures. The period of culture was 10 days. The error bars represented standard deviations, (*n* = 3); Different letters (*i.e.*, a–s) indicated significant differences among the treatments in each YPS addition time at *p* = 0.05 level.

Figure 3 shows the effects of yeast polysaccharide (YPS) on the growth and flavonoids accumulation of *F. tataricum* sprout cultures, which were dependent on both YPS concentration and its treatment period. As seen from Figure 3A, compared with the control culture of 0.92 gdw/100 sprouts, with early treatment, the sprout biomass decreased by about 2–10% with 100–800 mg/L YPS applied on day 0, but a slightly increase with the late treatment (days 3, 6 and 9). 

**Figure 4:**
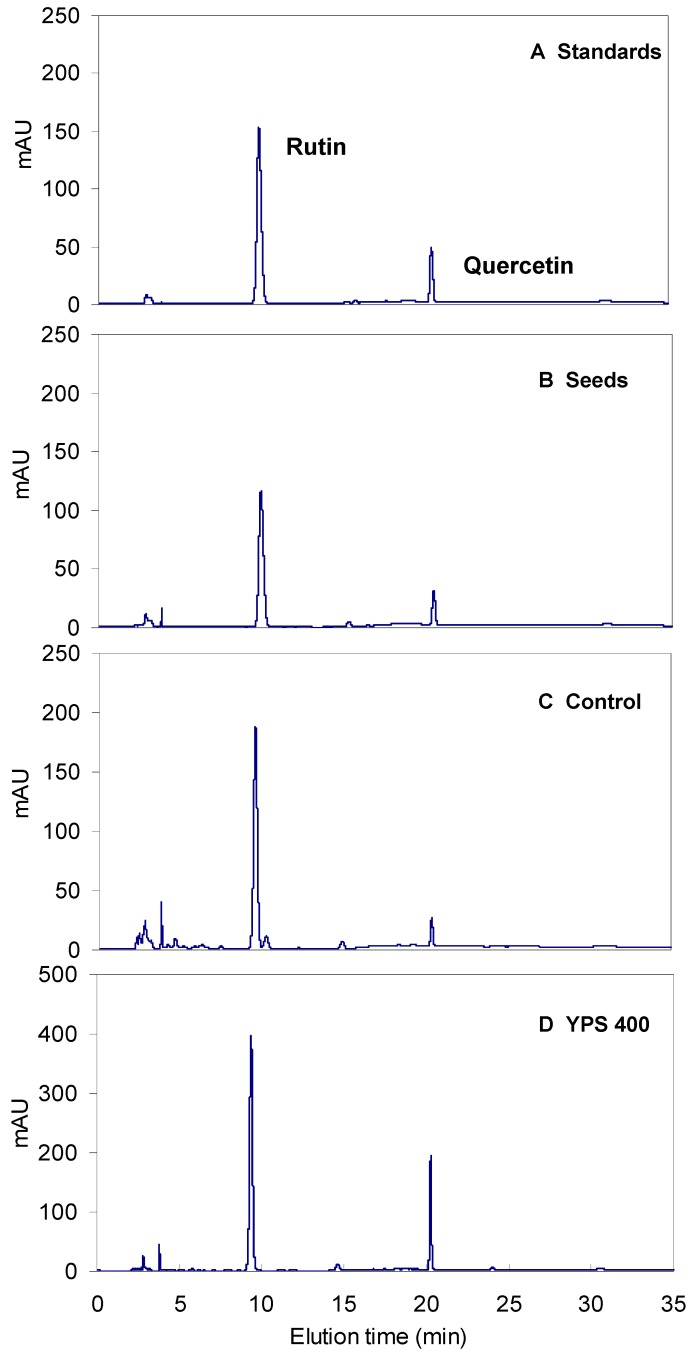
HPLC profiles for flavonoids analysis: rutin and quercetin standards (**A**), buckwheat seeds of Xiqiao-01 (**B**), control sprout cultures (**C**), and YPS (400 mg/L) treated sprouts (**D**).

With 800 mg/L YPS applied on day 9 of the cultures, the sprout biomass was increased by more than 8% in comparison with the control culture (0.99 gdw/ 100 sprouts *versus* 0.92 gdw/ 100 sprouts). Regarding rutin and quercetin accumulation, they were effectively stimulated by the YPS, most dramatically with 400 mg/L of YPS applied on day 6, and the total flavonoids content was 42.8 mg/g, or about 1.4-fold more in comparison with the control of 31.2 mg/g (Figure 3B). Correspondingly, the total flavonoids yield was as high as 20.7 mg/100 sprouts, about 1.5-fold compared to that of the control 14.3 mg/100 sprouts (Figure 3C. The typical HPLC profiles of rutin and quercetin standards, buckwheat seeds, control sprout cultures, and YPS treated sprout samples are shown in Figure 4.

### 2.3. Kinetics of F. tataricum Sprout Growth and Flavonoid Accumulation after Treatment with YPS

**Figure 5:**
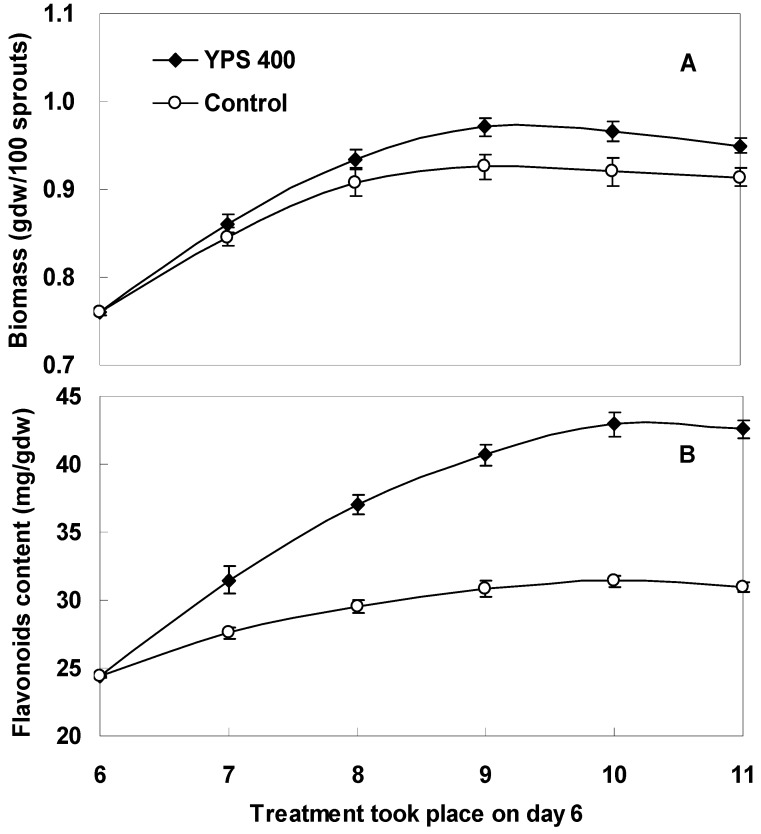
Kinetic studies of biomass growth (**A**) and flavonoids accumulation (**B**) of *F. tataricum* sprout cultures after treatment with 400 mg/L of YPS. YPS was applied to the sprout cultures on day 6, and the total period of culture was 11 days; The error bars represented standard deviations, (*n* = 3).

According to the previous investigation, the maximal flavonoids accumulation was obtained when the sprouts were treated with YPS at 400 mg/L on day 6 (Figure 3). Therefore, the kinetic studies of biomass growth and flavonoids accumulation in *F. tataricum* sprout cultures elicited by YPS (400 mg/L) were further studied, which are shown in Figure 5. The promoting effect of YPS on the sprout growth of *F. tataricum* could be noticed after 2 days treatment (Figure 5A. The maximum sprout biomass was 0.97 gdw/100 sprouts obtained on day 9, about 1.1-fold compared to that of the control 0.92 gdw/100 sprouts. The stimulation effect of YPS on flavonoids production of *F. tataricum* sprouts could be notably observed after 1 day treatment, and then followed a steady increase to the end of the culture period. The highest flavonoids content of the sprouts was 43.0 mg/gdw, about 1.4-fold in comparison with the control of 31.4 mg/gdw (Figure 5B). 

**Figure 6:**
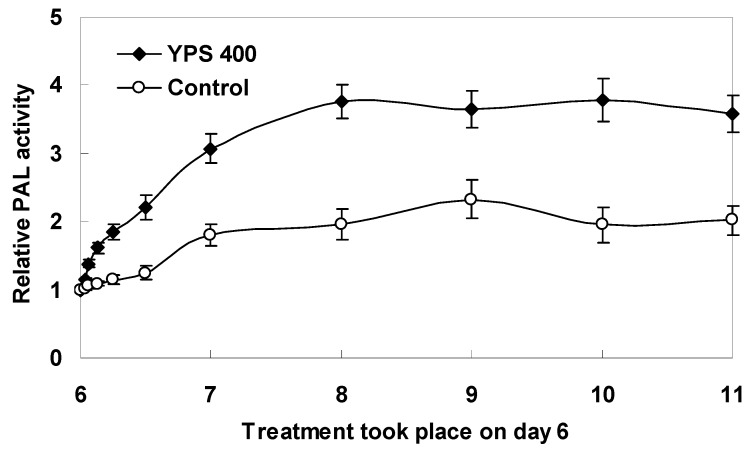
Time courses of PAL activity of *F. tataricum* sprouts after elicitation treatment with 400 mg/L of YPS in comparison with the control. YPS was applied to the sprout cultures on day 6, and the period of culture was 11 days; The error bars represented standard deviations, (*n* = 3).

As shown in Figure 6, the intracellular PAL activity of *F. tataricum* sprout cells was significantly stimulated by the YPS, from 1.3- to 1.9-fold of the control level over 5 days. PAL is a key enzyme at the entrance step in the phenylpropanoid pathway in plants, and its activity increase stimulated by the elicitors is suggestive of an enhanced secondary metabolism in the plant cells [15,23]. On the basis of these results obtained in present study, it could be speculated that the phenylpropanoid pathway was closely associated with the flavonoids biosynthesis in *F. tataricum* sprout cultures. That was in general agreement with those found in previous reports [24,25,26]. Moreover, it also indicated that the exogenous yeast polysaccharide (YPS), as an efficient biotic elicitor, may be taken up by receptors on the surface of the sprouts or transformed to a stress signal in stimulating the functional metabolites accumulation of buckwheat sprouts. Anyway, these valuable results provide further evidence for the elicitor activity of YPS in stimulating the stress responses and secondary metabolism of *F. tataricum* sprout cultures. 

## 3. Experimental

### 3.1. Source and Cultivation of Buckwheat Sprouts

Buckwheat seeds (cultivar Xiqiao-01 of *Fagopyrum tataricum*) were obtained from the Coarse Cereal Processing Center (CCPC) of Chengdu University (Chengdu, Sichuan, China). Healthy buckwheat seeds were surface-sterilized for 5 min in 2.5% sodium hypochlorite solution followed by a quick deionized-H_2_O rinse for three times (*i.e.*, 1 min each time). Then, the seeds were soaked in sterilized water at 25 °C for 2 h prior to placement into a commercial germination box (100 seeds in each box). Each germination box (120 × 120 × 50 mm) was lined with three autoclaved filter papers (HT4X12*12) moistened with 10 mL distilled H_2_O. The buckwheat sprouts were cultivated in a illumination incubators with 25 ± 1 °C and 75% relative humidity for 10–12 days. 

### 3.2. Preparation and Application of YPS

The yeast extract (YE, Y4250) was purchased from Sigma (St. Louis, MO, USA). The yeast polysaccharide (YPS) was the polysaccharide fraction of YE precipitated by ethanol as described previously [23]. Briefly, YE (20 g) was dissolved in distilled water (100 mL) and then mixed with ethanol (400 mL), and allowed to precipitate for 4 days at 4 °C in a refrigerator. The crude polysaccharide fraction was further purified by another round of ethanol precipitation. The final gummy precipitate was dissolved in distilled water (50 mL) and stored at 4 °C prior to use. The concentration of YPS was determined by the anthrone test using glucose as a reference [27]. 

YPS was applied to the *F. tataricum* sprout cultures at the following five concentrations (50, 100, 200, 400 and 800 mg/L) on days 0, 3, 6 and 9 of culture, respectively. The *F. tataricum* sprout cultures were harvested on day 10 for measurement of their biomass and flavonoids content. After the preliminary experiments, 400 mg/L of YPS was examined to be the most effective elicitation treatment, and it was applied in the next experiments on the time courses of YPS-treated sprout growth and flavonoids accumulation in *F. tataricum* sprout cultures.

### 3.3. Measurement of Biomass and Flavonoids Content

The sprouts of *F. tataricum* were harvested from the germination box and rinsed thoroughly with distilled water, and blotted dry by paper towels to obtain the fresh weight (fw), and then dried at 40–45 °C in an oven to attain the constant dry weight (dw). The dried spouts were ground into powder, passed through the screens, and the powder passing between 20–40 mesh screens was selected. The extraction was performed by mixing sample (0.1 g) with methanol-water (25 mL, 70%, v/v) solution in a conical flask under sonication for 30 min. After removal of the solid, the filtrates were transferred into a 25 mL volumetric flask and the volume adjusted to 25 mL with 70% methanol. The contents of rutin and quercetin were analyzed by high-performance liquid chromatography (HPLC). The HPLC system was equipped with two LC-10ATvp pumps and a SPD-M10Avp diode-array detector (Shimadzu, Kyoto, Japan), and using a C_18_ column (4.6 mm × 250 mm, 5 μm, Phenomenex, Torrance, CA, USA). The separation was performed using a mixture of acetonitrile and distilled water (0.2% H_3_PO_4_) with a gradient elution: (0–8 min, 20% acetonitrile; 8–13 min, 20–40% acetonitrile; 13–29 min, 40% acetonitrile; 29–30 min, 40–20% acetonitrile; 30–35 min, 20% acetonitrile). The flow rate was set at 1.0 mL/min, and UV detection at 365 nm. The temperature of the column was set at 30 °C, and the sample injection volume was 20 μL. The rutin and quercetin were detected and quantified with the standards obtained from the Institute for Identification of Pharmaceutical and Biological Products (Beijing, China). 

### 3.4. Measurement of PAL Activity

The phenylalanine ammonia lyase (PAL) was extracted from the fresh *F. tataricum* sprouts with borate buffer (pH 8.8). The sprout cells were ground in the buffer (0.2 g/mL) for 2 min with a pestle and mortar on ice, and then centrifuged at 8000 rpm and 4 °C for 20 min to obtain a solid-free extract. The PAL activity was determined based on the conversion of L-phenylalanine to cinnamic acid by the method of Wu and Lin [28]. 

### 3.5. Statistical Analysis

All treatments were performed in triplicate, and the results were represented by their mean values and the standard deviations (SD). The data were submitted to analysis of variance (one-way ANOVA) to detect significant differences by PROC ANOVA of SAS version 8.2. The term significant has been used to denote the differences for which *p* ≤ 0.05.

## 4. Conclusions

This is the first report on the effects of yeast polysaccharide (YPS) on growth and flavonoid accumulation in *F. tataricum* sprout cultures. Without obvious change in the appearance of the sprouts, the exogenous YPS effectively stimulated the flavonoids production of *F. tataricum* sprouts, and the stimulation effect was concentration-dependent. With application of 400 mg/L of YPS to the sprout cultures on day 6, the total rutin and quercentin content was effectively increased to 42.8 mg/gdw, or about 1.4-fold more in comparison with the control of 31.2 mg/gdw. The present study also revealed that the accumulation of these bioactive metabolites was caused by the stimulation of the phenylpropanoid pathway by YPS treatment. Although, there are still many issues such as the chemical composition of yeast polysaccharide, the structure-activity relationship, the physiological responses and biochemical reactions of the sprouts induced by the YPS, need to be further clarified and resolved. The results from this study suggest that it could be an effective strategy for improving the functional quality of the *F. tataricum* sprouts provided with YPS. As yeast polysaccharide is commercially available or can be readily prepared and easily administered to the sprout cultures, it should be helpful for practical application in the laboratory or large-scale production of nutritional buckwheat sprouts in the future.
